# Increased interleukin-6 is associated with long COVID-19: a systematic review and meta-analysis

**DOI:** 10.1186/s40249-023-01086-z

**Published:** 2023-04-24

**Authors:** Jing-Xian Yin, Yannick Luther Agbana, Zhi-Shan Sun, Si-Wei Fei, Han-Qing Zhao, Xiao-Nong Zhou, Jun-Hu Chen, Kokouvi Kassegne

**Affiliations:** 1 School of Global Health, Chinese Centre for Tropical Diseases Research, Shanghai Jiao Tong University School of Medicine, Shanghai, 200025 People’s Republic of China; 2 Pan African University Life and Earth Sciences Institute (PAULESI), University of Ibadan, Ibadan, Nigeria; 3 National Institute of Parasitic Diseases at Chinese Centre for Disease Control and Prevention (Chinese Centre for Tropical Diseases Research), National Health Commission of the People’s Republic of China (NHC) Key Laboratory of Parasite and Vector Biology, World Health Organization (WHO) Collaborating Centre for Tropical Diseases, National Centre for International Research On Tropical Diseases of the Chinese Ministry of Science and Technology, Shanghai, 200025 People’s Republic of China

**Keywords:** SARS-CoV-2, COVID-19, Interleukin-6, Long COVID-19, Immune mediator, Meta-analysis

## Abstract

**Background:**

Coronavirus disease 2019 (COVID-19) can involve persistence, sequelae, and other clinical complications that last weeks to months to evolve into long COVID-19. Exploratory studies have suggested that interleukin-6 (IL-6) is related to COVID-19; however, the correlation between IL-6 and long COVID-19 is unknown. We designed a systematic review and meta-analysis to assess the relationship between IL-6 levels and long COVID-19.

**Methods:**

Databases were systematically searched for articles with data on long COVID-19 and IL-6 levels published before September 2022. A total of 22 published studies were eligible for inclusion following the PRISMA guidelines. Analysis of data was undertaken by using Cochran's Q test and the Higgins I-squared (*I*^2^) statistic for heterogeneity. Random-effect meta-analyses were conducted to pool the IL-6 levels of long COVID-19 patients and to compare the differences in IL-6 levels among the long COVID-19, healthy, non-postacute sequelae of severe acute respiratory syndrome coronavirus 2 (SARS-CoV-2) infection (non-PASC), and acute COVID-19 populations. The funnel plot and Egger's test were used to assess potential publication bias. Sensitivity analysis was used to test the stability of the results.

**Results:**

An increase in IL-6 levels was observed after SARS-CoV-2 infection. The pooled estimate of IL-6 revealed a mean value of 20.92 pg/ml (95% *CI* = 9.30–32.54 pg/ml, *I*^2^ = 100%, *P* < 0.01) for long COVID-19 patients. The forest plot showed high levels of IL-6 for long COVID-19 compared with healthy controls (mean difference = 9.75 pg/ml, 95% *CI* = 5.75–13.75 pg/ml,* I*^2^ = 100%, *P* < 0.00001) and PASC category (mean difference = 3.32 pg/ml, 95% *CI* = 0.22–6.42 pg/ml,* I*^2^ = 88%, *P* = 0.04). The symmetry of the funnel plots was not obvious, and Egger’s test showed that there was no significant small study effect in all groups.

**Conclusions:**

This study showed that increased IL-6 correlates with long COVID-19. Such an informative revelation suggests IL-6 as a basic determinant to predict long COVID-19 or at least inform on the “early stage” of long COVID-19.

**Supplementary Information:**

The online version contains supplementary material available at 10.1186/s40249-023-01086-z.

## Background

The newly emerged coronavirus of likely bat origin, severe acute respiratory syndrome coronavirus 2 (SARS-CoV-2), causes a disease known as coronavirus disease 2019 (COVID-19) [1, 2]. The disease has affected hundreds of millions of people along with the devastating global consequences of an unprecedented public health crisis. The clinical spectrum of COVID-19 ranges from asymptomatic infection to fatal disease [3, 4]. As of 30 August 2022, there have been more than 600 million confirmed cases of COVID-19, with an estimated six and a half million deaths [5, 6].

The term “long COVID” is commonly used to describe signs and symptoms that continue or develop after acute COVID-19 [7]. The National Institute for Health and Care Excellence (NICE, United Kingdom) defines long COVID as symptoms that continue or develop after acute COVID-19 infection and that cannot be explained by an alternative diagnosis. This term includes ongoing symptomatic COVID-19, from four to 12 weeks post infection, and post-COVID-19 syndrome, beyond 12 weeks post infection [3]. The National Institute of Health (NIH) uses the United States Centers for Disease Control and Prevention (CDC) definition of long COVID-19, which describes the condition as sequelae that extend beyond four weeks after initial infection [4]. Long COVID-19 is a matter of concern. It has been reported that long COVID-19 is associated with diverse potential complications, including postintensive care syndrome, postviral fatigue syndrome, long-term COVID-19 syndrome, and permanent organ damage [8], which have serious consequences that cannot be ignored. Moreover, any patient with COVID-19 may develop long COVID-19, regardless of the severity of infection and the intensity of the treatment received. Patients treated in wards and intensive care units (ICUs) showed little difference in the incidence of long-term symptoms associated with COVID-19 [9]. Additionally, a recent study reported that 60 days after disease onset, 87.1% of discharged patients with COVID-19 still experience at least one symptom, and 55% experience three or more symptoms, such as dyspnea, chest pain, fatigue, and reduced quality of life [10]. Thus, COVID-19 and long COVID-19 are closely related, and long COVID-19 is an integral part of COVID-19 treatment management.

A report describing the immunological profile of critically ill patients with COVID-19 suggested hyperactivation of the humoral immune pathway, including IL-6, as a critical mediator of respiratory failure, shock, and multiorgan dysfunction [11]. Very recently, a cohort study showed that acute COVID-19 or postacute sequelae of COVID-19 (PASC) are not related to autoantibodies but to elevated plasma levels of proinflammatory cytokines such as interleukin-1 beta (IL-1β), IL-6, and tumor necrosis factor alpha (TNF-α) [12]. Additionally, in a study on cytokine profiles in acute COVID-19 and long COVID-19 syndrome, Queiroz and colleagues reported that IL-6 is one of the important cytokines that is relevant to the outcome of COVID-19, including disease duration and severity [13]. IL-6 is generated at sites of infection and inflammation by immune cells [14], adipocytes [15], and endothelial cells [16]. The cytokine promotes the differentiation of naive CD4 + T cells, which suggests that it has an essential role in the development of adaptive immunity [14]. IL-6 has been identified as a potential mediator of long-term neuropsychiatric symptoms of COVID-19 [17]. B cells from patients with acute COVID-19 displayed an IL-6 cytokine imbalance in response to Toll-like receptor activation, skewed toward a proinflammatory phenotype [18].

The potential for the hyperactivation of IL-6 in the host immune pathway contributes to the development of long-term symptoms of COVID-19. Therefore, characterizing such immune dysregulation is a research priority. We designed a systematic review and meta-analysis to explore correlates of IL-6 levels and long COVID-19 for future trials targeting this immune mediator.

## Methods

### Literature search strategies

This study followed the Preferred Reporting Items for Systematic Reviews and Meta-Analyses (PRISMA) guidelines [19–22].

Relevant studies published before September 2022 were systematically searched online in the PubMed, EMBASE, Web of Science, and Cochrane Library databases. The following search terms were used in different combinations: (“long COVID” OR “chronic COVID-19” OR “COVID long-hauler” OR “COVID-19 long-hauler” OR “long haul COVID” OR “long hauler COVID” OR “post-acute COVID-19”) AND (“Interleukin-6” OR “IL6” OR “IL-6” OR “B-Cell Stimulatory Factor 2” OR “B-Cell Stimulatory Factor-2” OR “B-Cell Differentiation Factor-2” OR “B Cell Differentiation Factor 2” OR “BSF-2” OR “Hybridoma Growth Factor” OR “IFN-beta 2” OR “Interferon beta-2” OR “Interferon beta 2” OR “Plasmacytoma Growth Factor” OR “Hepatocyte-Stimulating Factor” OR “Hepatocyte Stimulating Factor” OR “MGI-2” OR “Myeloid Differentiation-Inducing Protein” OR "Myeloid Differentiation Inducing Protein”). References of full-text articles were also searched for pertinent studies that discussed the connection between IL-6 levels and long COVID-19. There were no language restrictions.

### Definition

Long COVID-19, also known as postacute sequelae of COVID-19 (PASC), is the term used to describe the subset of patients recovering from COVID-19 who develop a wide range of persistent symptoms that do not resolve after several weeks to months. Long COVID-19 is not associated with disease severity [23, 24].

### Inclusion and exclusion criteria

The eligibility requirements were as follows: (1) the study subjects were COVID-19 patients in the post-COVID-19 phase, as well as healthy individuals; (2) the studies primarily investigated levels of IL-6 in long COVID-19; (3) patients were clearly separated into different groups, namely, the long COVID-19 group, the non-PASC group, the acute COVID-19 group, and the healthy individuals’ group; (4) the IL-6 levels were measured quantitatively; and (5) sufficient information and data were provided to calculate and estimate the mean and standard deviation (*SD*) values. The exclusion criteria were as follows: (1) duplicate publications; (2) reviews, meta-analyses, protocols, editorials, letters, preprints, and unavailable full texts; (3) studies without patient samples; and (4) studies that did not report IL-6 values in long COVID-19.

### Publication quality assessment

The quality of the included studies was assessed using the Newcastle–Ottawa Scale (NOS). Three main categories were considered: selection, comparability, and outcome. In addition, the stars rating system was used [25]. The NOS scores ranged from zero stars (lowest score) to nine stars (highest score). A study with a NOS score > 5 was considered a high-quality study [20].

### Data extraction

Three investigators independently reviewed all eligible studies and extracted the following information: the first author's name, year of publication, sample size, detection method of IL-6, mean, and *SD*. Any controversial issue was resolved by discussion. Where the mean and *SD*s are not provided, they were estimated using Wan and colleagues' method [26] or the calculator in Review Manager 5.4.1. program (https://community.cochrane.org/help/tools-and-software/revman-5/revman-5-download/). Where the IL-6 value for healthy individuals was not provided, the normal value of IL-6 as reported in the meta-analysis study by Said and colleagues [27] was used.

### Analysis of data

Count data and nominal variables from original studies are presented as proportions with percentages, while continuous data are presented as means ± *SD* or medians and interquartile range (IQR).

For data deemed adequately homogenous in terms of patient characteristics, interventions, and clinical outcomes, meta-analyses were undertaken using random-effect models. For statistical homogeneity, medians and IQRs were converted to means with *SD*s to maximize the number of studies eligible for meta-analysis [26]. Pooled estimates of IL-6, 95% confidence intervals (95% *CIs*) and forest plots were assessed [28, 29].

To assess the association between IL-6 levels and the different clinical groups, including long COVID-19, non-PASC, acute COVID-19 and healthy individuals, Review Manager 5.4.1 (The Nordic Cochrane Centre, The Cochrane Collaboration, Copenhagen) was used for all statistical analyses. The pooled means were used to assess the relationship between IL-6 levels and long COVID-19. Cochran’s Q test and the Higgins I-squared (*I*^2^) statistic were used to examine the heterogeneity of the study results. The fixed-effect model was used when there was no heterogeneity (*P* ≥ 0.10 and/or* I*^2^ < 50%); otherwise, the random-effect model was used.

To explore the potential source of heterogeneity, subgroup analyses were carried out based on the main relevant variables. According to the study design, five subgroups were generated for analysis, including cohort studies, case–control studies, cross-sectional studies, randomized controlled trials, and case reports. In addition, two subgroups, including direct mean and indirect mean, were compared according to the data extraction method.

Funnel plots and Egger's tests were performed to assess potential publication bias using R studio 4.1.3 (R Foundation for Statistical Computing, Austria). Sensitivity analysis was also performed using R studio to assess the impact of individual studies on the pooled effect [30]. A *P*-value of less than 0.05 was deemed statistically significant.

## Results

### Characteristics of the included studies

**Fig. 1:**
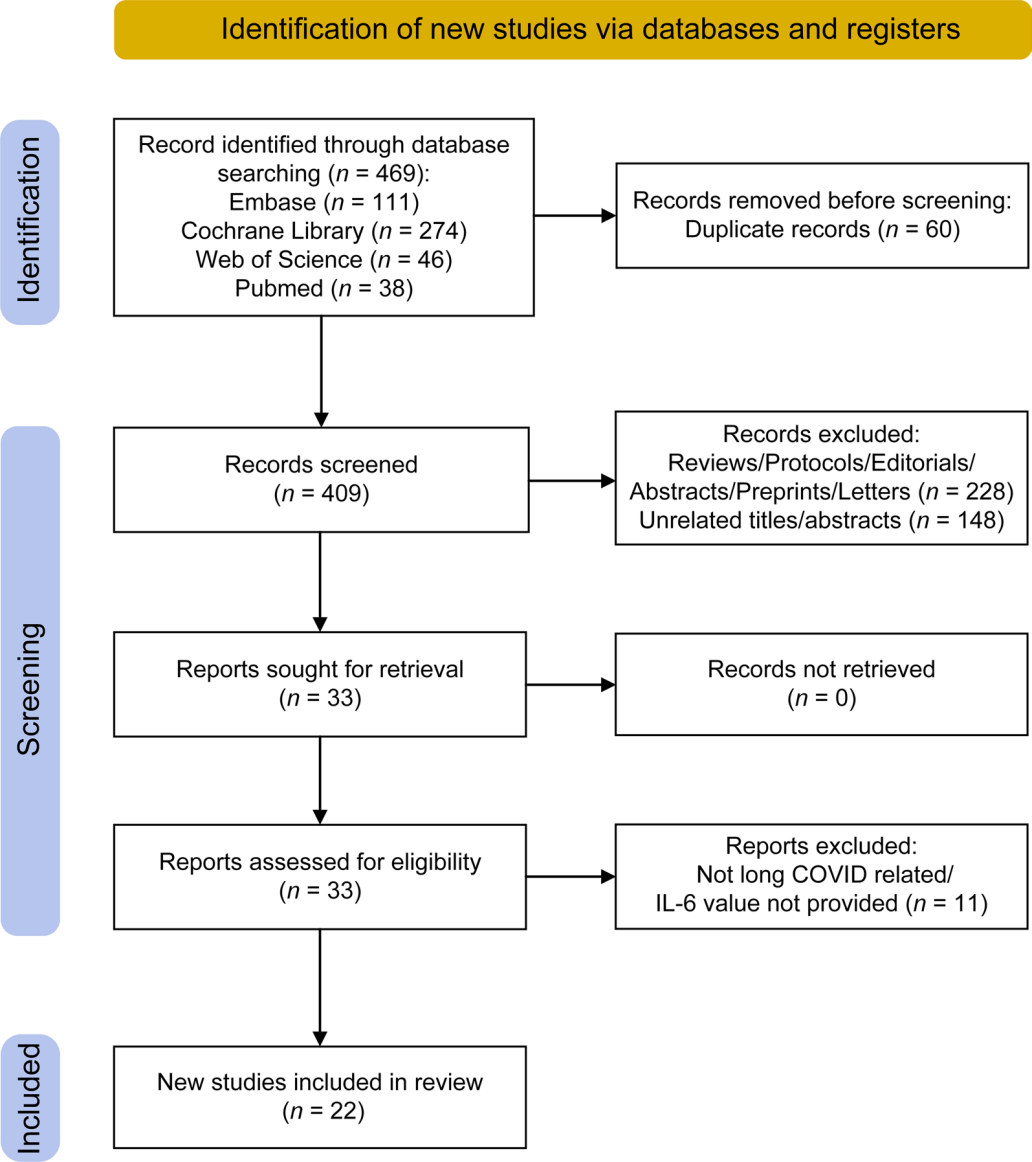
Flowchart of the literature search strategy. The flow diagram was generated based on the PRISMA 2020 guidelines (https://estech.shinyapps.io/prisma_flowdiagram/). The checklist for the flow diagram is provided in Additional file 2

A flowchart of the literature search strategy is shown in Fig. 1. According to the described search strategy, 469 articles were retrieved (Embase: 111 articles, Cochrane Library: 274 articles, Web of Science: 46 articles, and PubMed: 38 articles). A total of 60 articles were excluded for duplication, while the remaining 409 articles underwent an initial screening. On the basis of the type of article, title, and abstract, 376 articles were excluded, and a total of 33 full-text articles were assessed for eligibility. Eleven articles that did not relate to long COVID-19 or with an IL-6 value not provided were excluded. Finally, a total of 22 studies were retained for this work, 16 of which were used in the meta-analyses (Table 1). A total of 21/22 included studies were of high quality (NOS scores > 5), while only one study had a NOS score = 5. Information on the quality assessment reflected in NOS scores and the overall characteristics of the 22 included studies are summarized in Additional file 1: Tables S1 and S2, respectively.

**Table 1:** List of the studies used for the analysis of IL-6 levels in clinical COVID-19 and healthy individual groups

Study or Subgroup	Long COVID-19			Healthy control			Acute COVID-19			Non-PASC			D or I	St. Desi
	Mean	*SD*	*N*	Mean	*SD*	*N*	Mean	*SD*	*N*	Mean	*SD*	*N*		
Acosta-Ampudia et al., 2022 [37]	31.88	31.64	12	3.83	4.01	8	64.75	101.40	60	NR	NR	NR	I	Coh
Barros et al., 2021 [36]	40.77	55.46	118	5.186˟	15.93	3166	NR	NR	NR	NR	NR	NR	I	RCT
Cervia et al., 2022 [38]	8.77	14.71	85	0.53	0.85	40	3.8	7.03	49	NR	NR	NR	I	Coh
Colarusso et al., 2021 [39]	141.75	83.47	52	280.75	195.41	17	NR	NR	NR	NR	NR	NR	I	Cros
Dugani et al., 2022 [40]	8.6	23.98	69	2.95	8.03	66	NR	NR	NR	NR	NR	NR	I	Cc
Ganesh et al., 2021 [41]	2.2	0.28	108	0.45	0.36	108	NR	NR	NR	NR	NR	NR	I	Cros
Karosanidze et al., 2022 [42]	5.883	3.919	50	5.186˟	15.93	3166	NR	NR	NR	NR	NR	NR	D	RCT
Littlefield et al., 2022 [43]	1.55	2.83	20	5.186˟	15.93	3166	NR	NR	NR	0.87	1.59	20	I	Coh
Montefusco et al., 2021 [44]	0.05	0.15	10	0.3	0.34	14	3.22	3.69	8	NR	NR	NR	D	Coh
Patterson et al., 2021 [34]	20.47	66.75	121	2.21	1.54	29	76.45	NR	74	NR	NR	NR	I	Coh
Peluso et al., 2021(a) [45]	11.75	39.52	73	5.186˟	15.93	3166	NR	NR	NR	8.91	8.08	48	D	Coh
Peluso et al., 2021(b) [45]	21.38	23.36	73	5.186˟	15.93	3166	NR	NR	NR	6.31	6.37	48	D	Coh
Queiroz et al., 2022 [13]	19.29	10.81	225	5.186˟	15.93	3166	47.235	32.50	92	NR	NR	NR	I	Coh
Taha et al., 2021 [35]	25.52	3.45	100	5.186˟	15.93	3166	NR	NR	NR	NR	NR	NR	D	Cros
Townsend et al., 2021 [46]	2.32	2.4	3	5.186˟	15.93	3166	17.06	21.80	22	NR	NR	NR	I	Coh
Vyas et al., 2021 [47]	35.91	0.0036	1	5.186˟	15.93	3166	NR	NR	NR	NR	NR	NR	D	Cr
Wechsler et al., 2021 [48]	1.76	1.1	13	0.71	0.48	20	19.31	19.35	19	1.06	0.56	13	I	Coh

Mean: mean value of IL-6 estimated in pg/ml

*, the normal value of IL-6 in healthy individuals according to a meta-analysis study [27], which was used for studies in which the IL-6 value for healthy individuals was not provided

*SD*: standard deviation of IL-6 values; *N*: number of participants for estimation in a study; (a) and (b), different cohorts from the same article; NR: not related or not reported; D: mean data extraction using the direct mean value of IL-6; I: mean data extraction using the indirect mean value of IL-6; St: Desi, study design; Coh: cohort study; Cc: case–control study; Cros: cross-sectional study; RCT: randomized controlled trial; Cr: case report

### IL-6 levels increase after SARS-CoV-2 infection

**Fig. 2:**
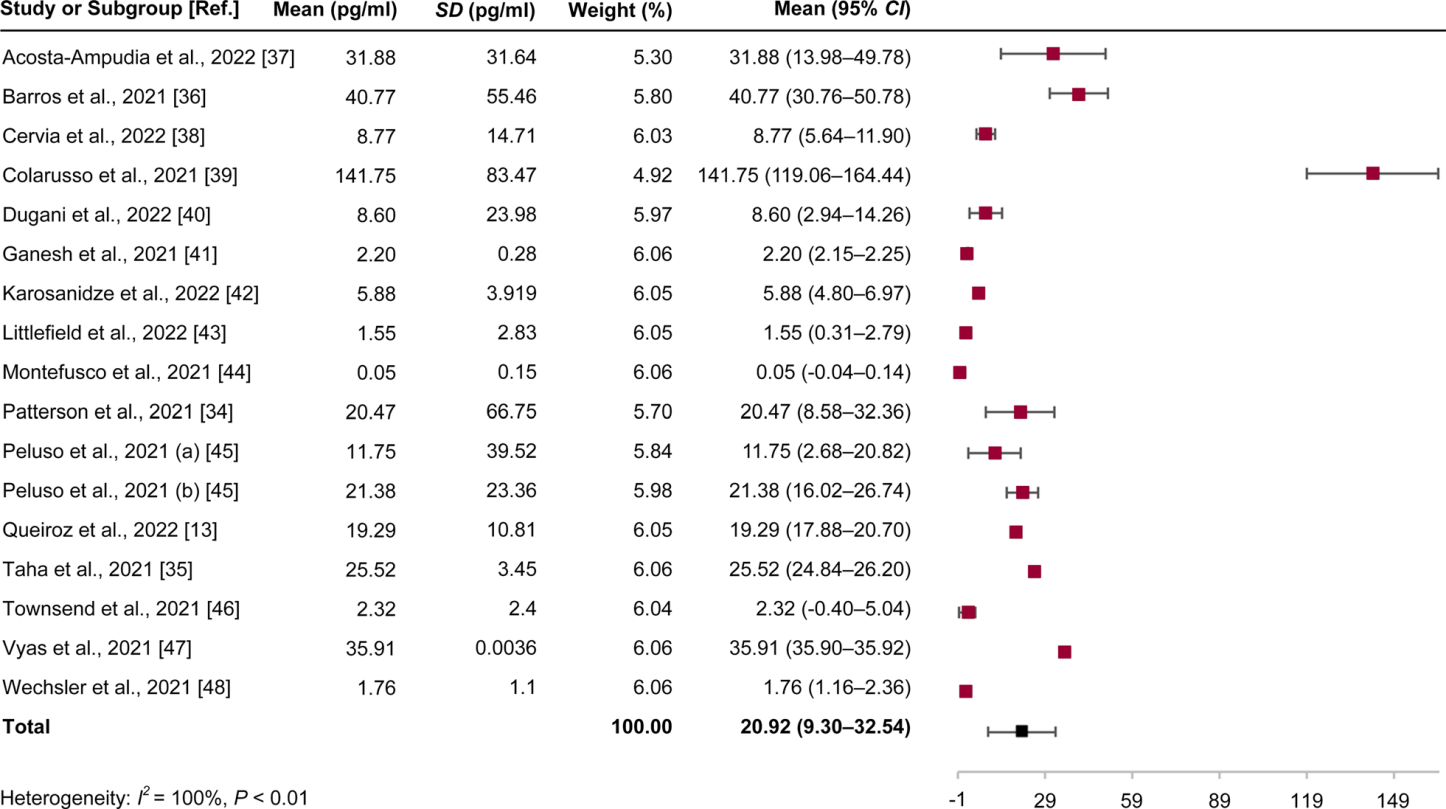
Pooled estimate of IL-6 for long COVID-19. Ref.: reference

**Fig. 3:**
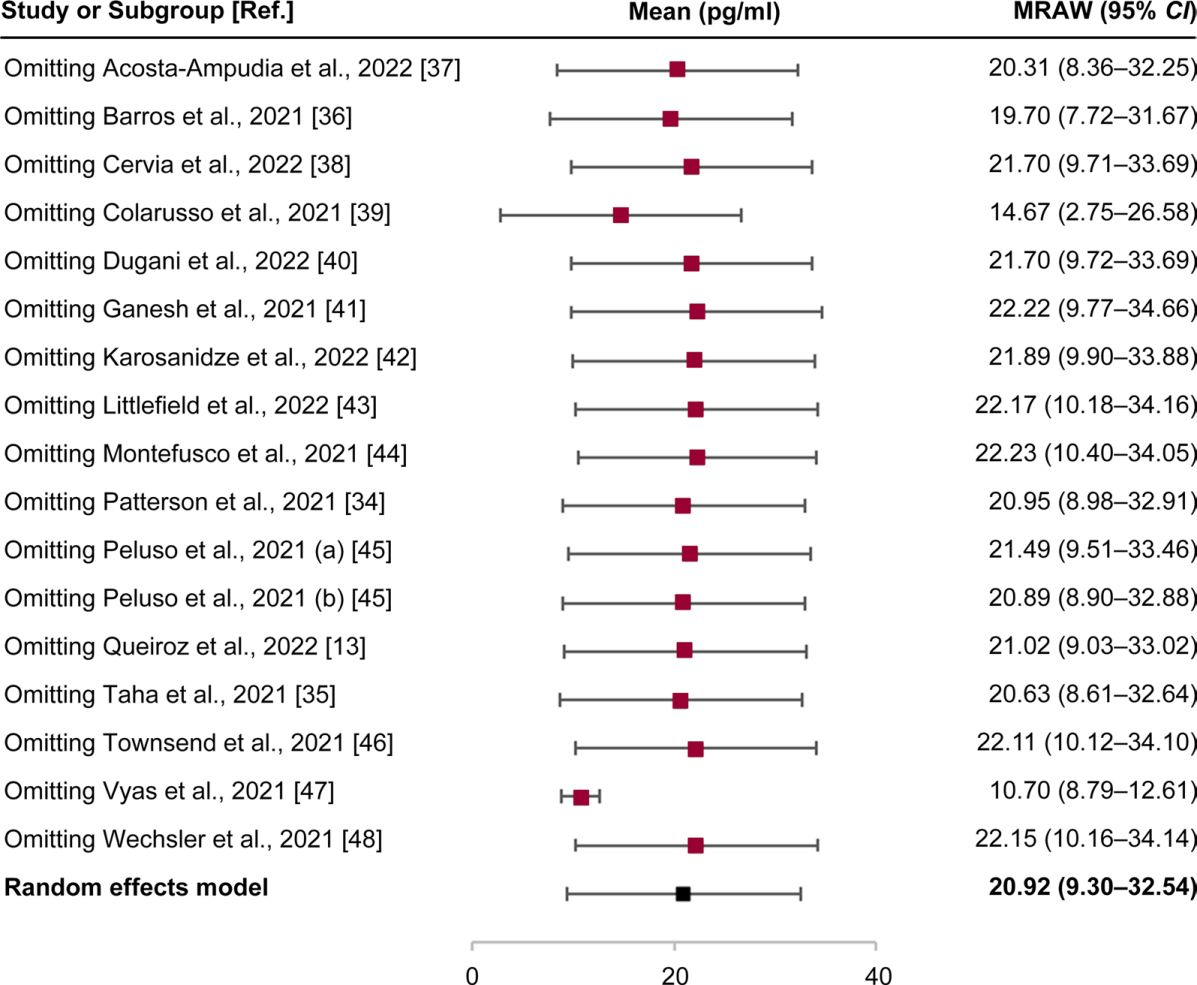
Sensitivity analysis of the pooled estimate of IL-6 for long COVID-19. Ref.: reference

Analysis of data from 17 cohorts of the 16 articles that were involved in this study allowed us to calculate the mean values of IL-6 in the study subjects, including the long COVID-19 group, acute COVID-19 group, and healthy individual group (Table 1). An increase in the levels of IL-6 after SARS-CoV-2 infection was observed. Collectively, this study considers and attributes 5.186 pg/ml as the mean value of IL-6 in healthy individuals [27] to 9 studies, which is obviously lower than that estimated for acute or long COVID-19 (Table 1). The pooled estimate of IL-6 in the long COVID-19 population from the included studies revealed a mean value of 20.92 pg/ml (95% *CI* = 9.30–32.54 pg/ml,* I*^*2*^ = 100%, *P* < 0.01) (Fig. 2), which was validated by the sensitivity analysis (Fig. 3). This implies that in COVID-19 patients, high levels of IL-6 (> 5.186 pg/ml) could be detected. Such evidence of an increase in levels of IL-6 is also associated with long COVID-19, which is defined after the time window of four weeks (≥ 28 days) post infection [13, 31–36].

### Increased interleukin-6 is associated with long COVID-19

**Fig. 4:**
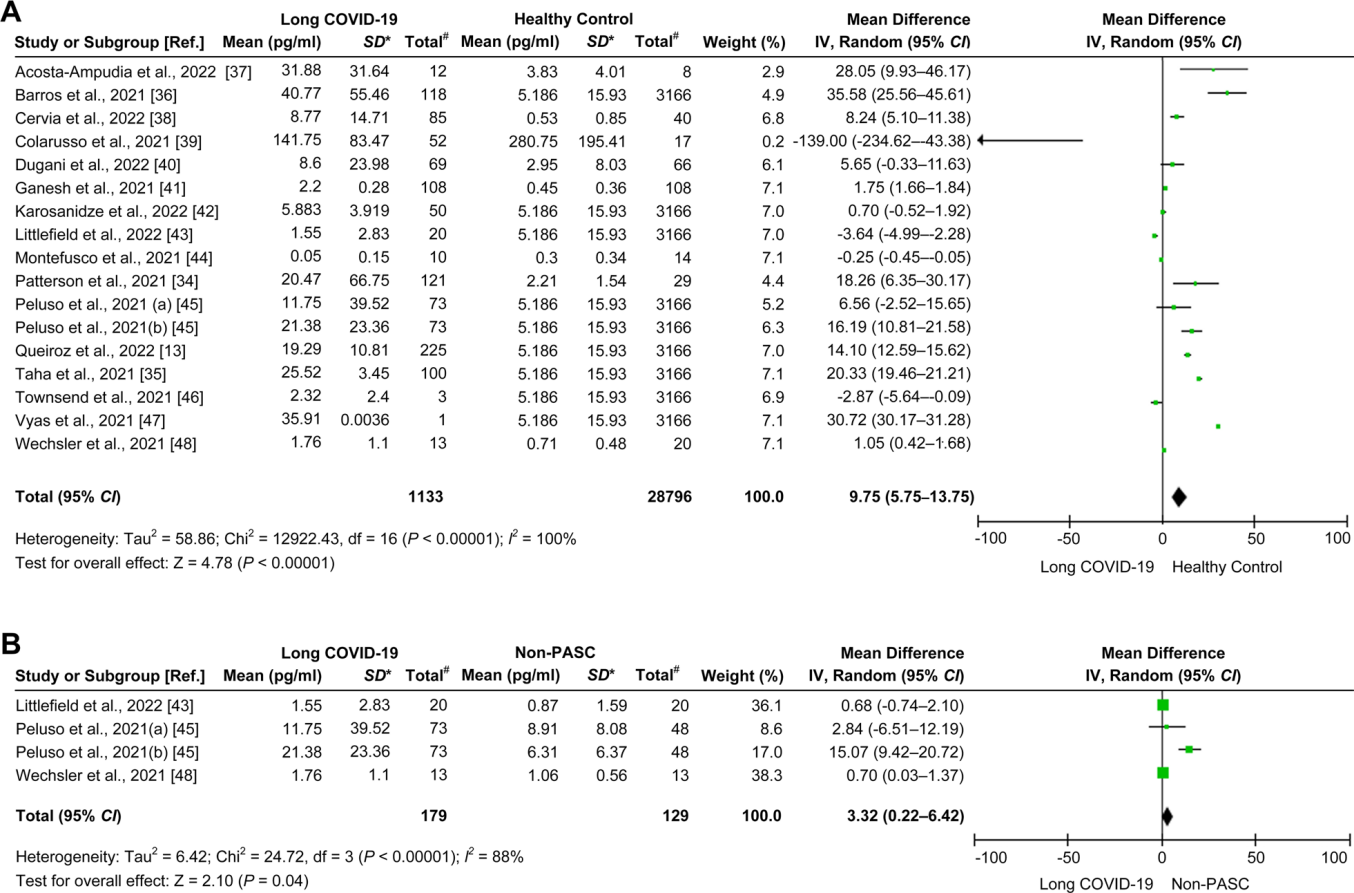
Comparison of the levels of IL-6 in the long COVID-19, non-PASC, and healthy individual groups. **A** Forest plot comparing long COVID-19 versus healthy individuals. **B** Forest plot comparing long COVID-19 versus non-PASC. Ref.: reference; *, standard deviation (pg/ml); #, total number of participants involved in the study or cohort

**Fig. 5:**
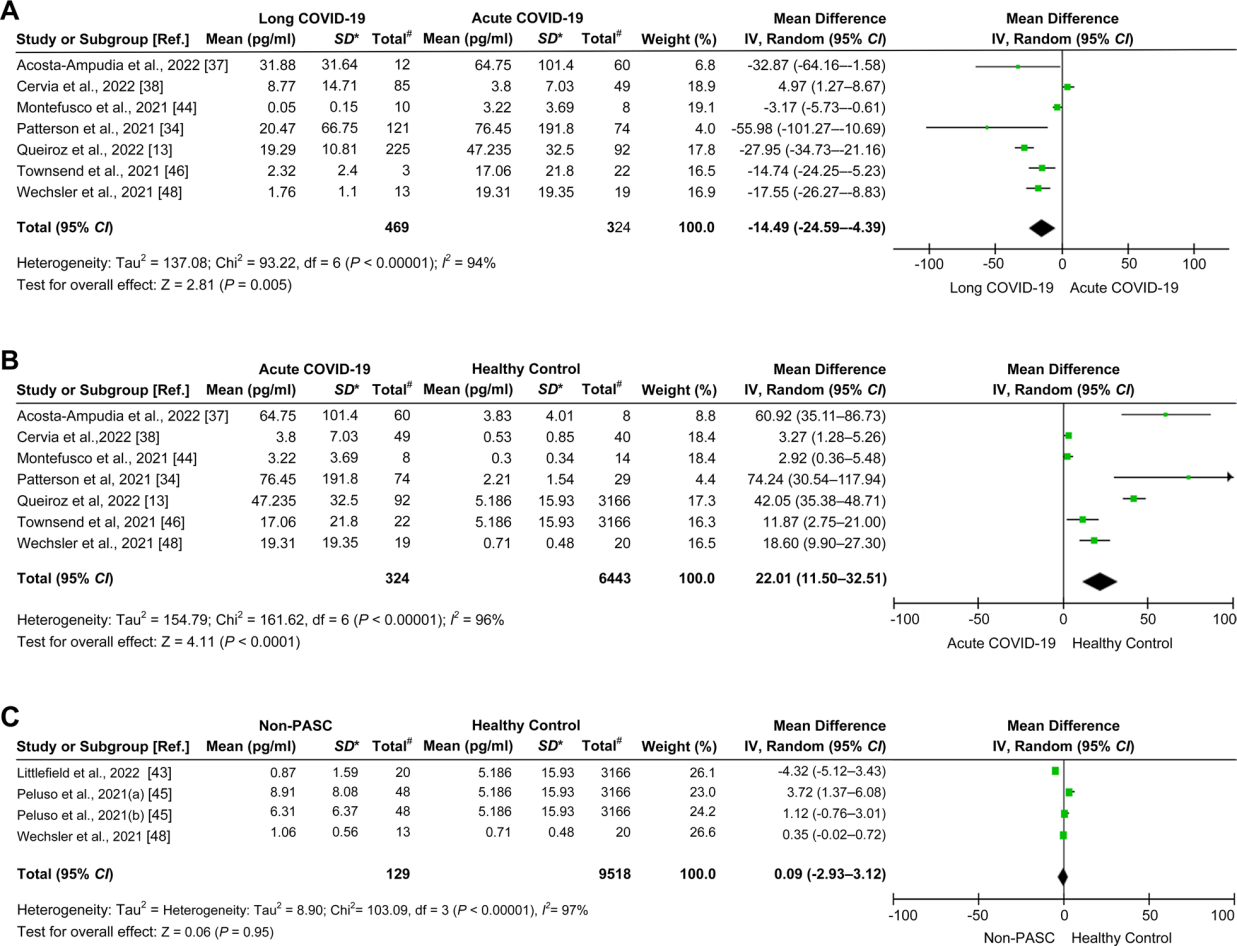
Comparison of levels of IL-6 in the long COVID-19, acute COVID-19, non-PASC, and healthy individual groups. **A** Forest plot comparing long COVID-19 versus acute COVID-19. **B** Forest plot comparing acute COVID-19 versus healthy individuals. **C** Forest plot comparing non-PASC versus healthy individuals. Ref.: reference; *, standard deviation (pg/ml); #, total number of participants involved in the study or cohort

The available data from 17 cohorts of 16 studies (shown in Table 1) were used to estimate differences in IL-6 levels between long COVID-19 patients and healthy individuals. Long COVID-19 patients had higher mean IL-6 levels than healthy individuals (mean difference = 9.75 pg/ml, 95% *CI* = 5.75–13.75 pg/ml,* I*^*2*^ = 100%, *P* < 0.00001; Fig. 4A). Likewise, when compared with the non-PASC group using four relevant cohorts from three studies [43, 45, 47], long COVID-19 patients had higher mean IL-6 levels (mean difference = 3.32 pg/ml, 95% *CI* = 0.22–6.42 pg/ml,* I*^2^ = 88%, *P* = 0.04; Fig. 4B). In contrast, when compared with patients in the acute phase using seven cohorts from seven studies [13, 34, 37, 38, 44, 46, 48], long COVID-19 patients had lower mean IL-6 levels (mean difference =  − 14.49 pg/ml, 95% *CI* =  − 24.59 to − 4.39 pg/ml,* I*^2^ = 94%, *P* = 0.005; Fig. 5A). Additional evaluation showed that the mean IL-6 levels in acute COVID-19 patients were higher than those in healthy individuals (mean difference = 22.01 pg/ml, 95% *CI* = 11.5–32.51 pg/ml,* I*^2^ = 96%, *P* < 0.0001; Fig. 5B). There was no statistically significant difference in IL-6 levels when comparing the non-PASC group and healthy individuals (mean difference = 0.09 pg/ml, 95% *CI* = − 2.93–3.12,* I*^2^ = 97%, *P* = 0.95; Fig. 5C). A summary of the meta-analysis results is shown in Table 2.

**Table 2:** Summary of meta-analysis results

Comparison	*P* value	Mean difference (pg/ml)	95% *CI* (pg/ml)	*I*^*2*^ (%)	Number of studies or cohorts for estimation
Long COVID-19 vs healthy control	< 0.00001	9.75	5.75–13.75	100	17
Long COVID-19 vs non-PASC	0.04	3.32	0.22–6.42	88	4
Long COVID-19 vs acute COVID-19	0.005	− 14.49	− 24.59 to − 4.39	94	7
Acute COVID-19 vs healthy control	< 0.0001	22.01	11.5–32.51	96	7
Non-PASC vs healthy control	0.95	0.09	− 2.93 to 3.12	97	4

*I*^2^: Higgins I-squared; *CI*: confidence interval

### Subgroup analysis

**Fig. 6:**
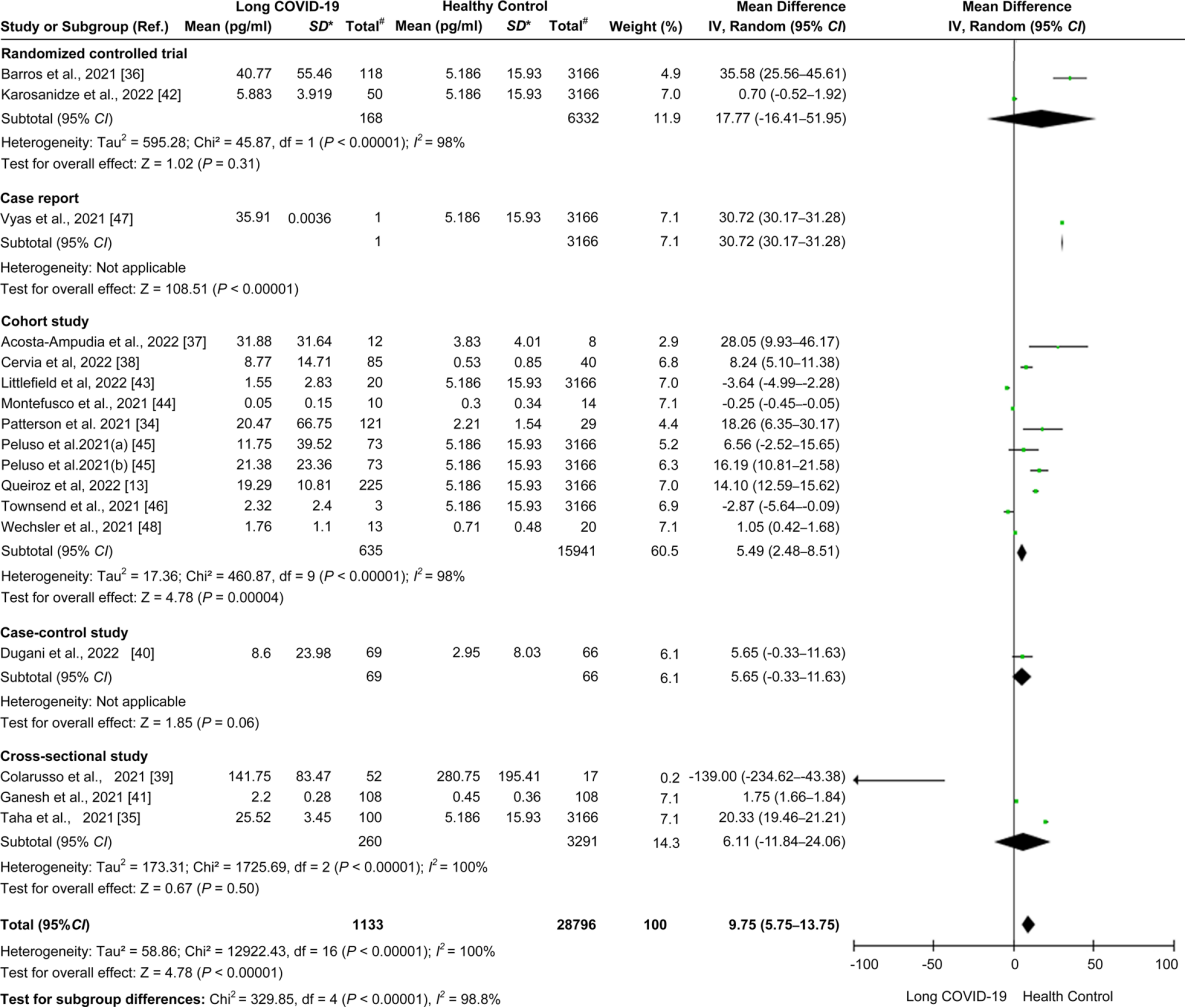
Subgroup analysis based on the study design. Forest plot comparing five different study designs. Ref.: reference; *, standard deviation (pg/ml); #, total number of participants involved in the study or cohort

The subgroup analysis based on different study designs is presented in Fig. 6. The level of IL-6 in long COVID-19 patients was higher than that in healthy individuals (mean difference = 5.49 pg/ml, 95% *CI* = 2.48–8.51, *P* < 0.01) in cohort studies, case–control studies (mean difference = 5.65 pg/ml, 95% *CI* =  − 0.33 to 11.63, *P* = 0.06), cross-sectional studies (mean difference = 6.11 pg/ml, 95% *CI* =  − 11.84 to 24.06, *P* = 0.5), case reports (mean difference = 30.72 pg/ml, 95% *CI* = 30.17–31.28, *P* < 0.00001), and RCTs (mean difference = 17.77 pg/ml, 95% *CI* =  − 16.41 to 51.95, *P* = 0.31).

**Fig. 7:**
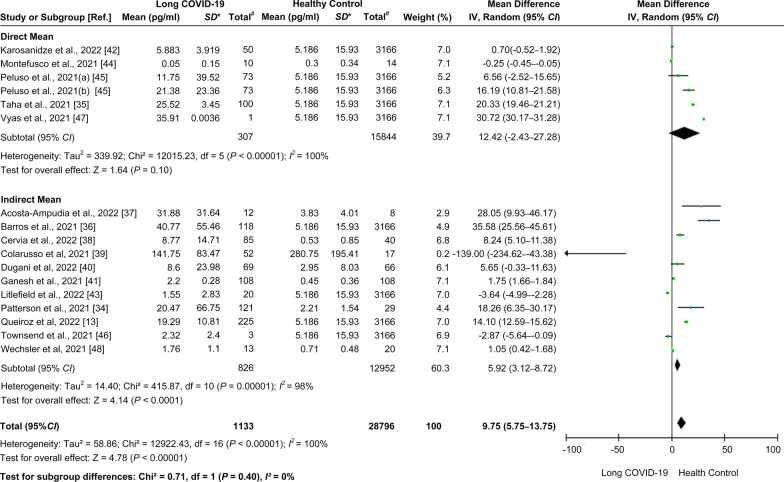
Subgroup analysis based on the data extraction method (direct mean or indirect mean). Forest plot comparing two different data extraction methods. Ref.: reference; *, standard deviation (pg/ml); #, total number of participants involved in the study or cohort

The subgroup analysis results of the data extraction method are illustrated in Fig. 7. The level of IL-6 in long COVID-19 patients was higher than that in healthy individuals, whether the mean values were given directly by the studies (mean difference = 12.42 pg/ml, 95% *CI* = − 2.43 to 27.28, *P* = 0.10) or obtained from the medians (mean difference = 5.92 pg/ml, 95% *CI* = 3.12–8.72, *P* < 0.0001).

### Publication bias

**Fig. 8:**
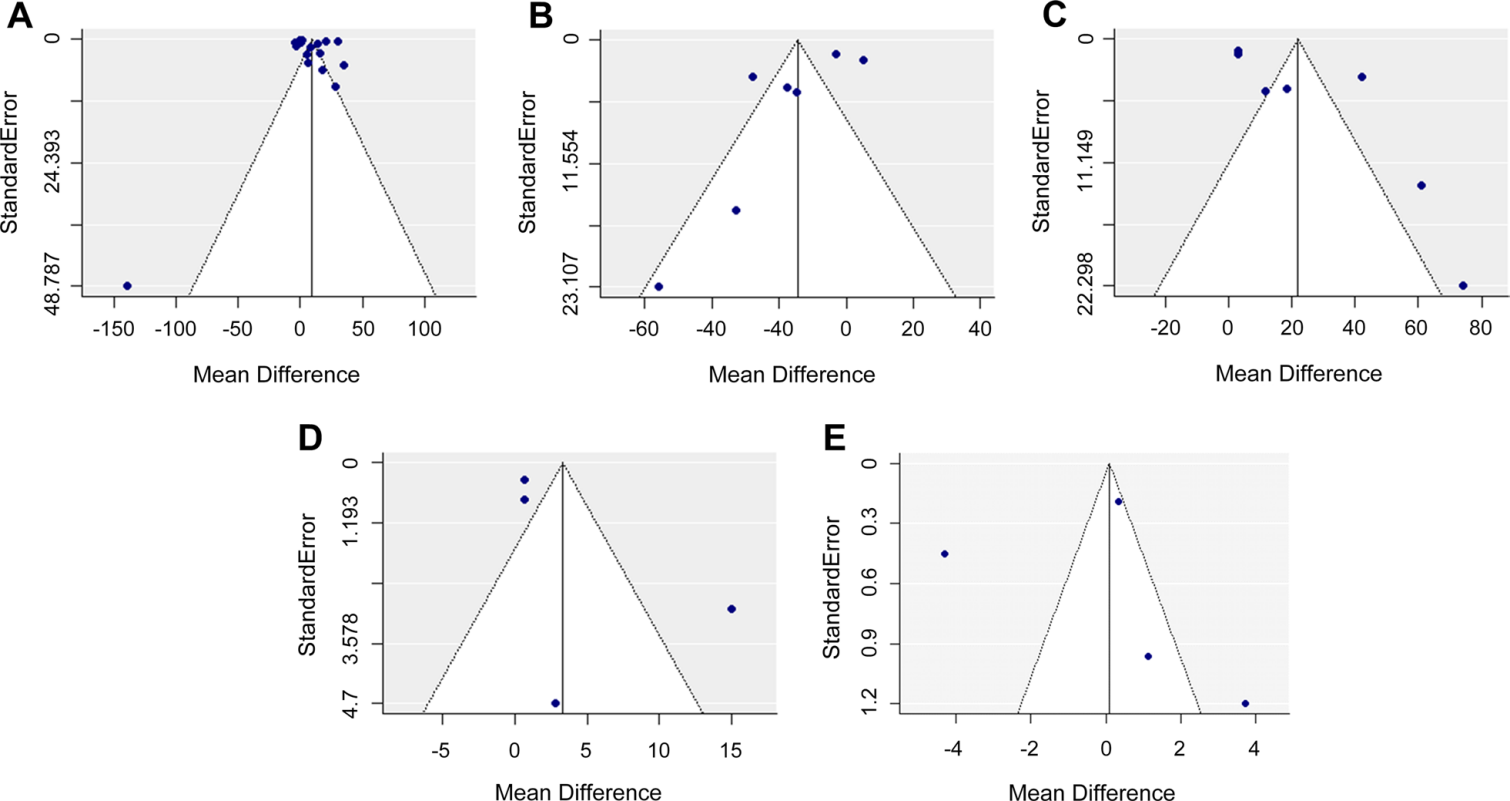
Publication bias analysis of the included studies. **A** Funnel plot of cohorts with long COVID-19 versus healthy individuals. **B** Funnel plot of cohorts with long COVID-19 versus acute COVID-19. **C** Funnel plot of cohorts with acute COVID-19 versus healthy individuals. **D** Funnel plot of cohorts with long COVID-19 versus non-PASC. **E** Funnel plot of cohorts with non-PASC versus healthy individuals

Funnel plots and Egger’s tests were used to determine potential publication bias (Fig. 8). The symmetry of the funnel plots was not obvious. The asymmetry of the plots may be due to heterogeneity. However, no publication bias was observed in any of the four groups. Egger’s test results showed that there was no significant small study effect in any of the four groups (long COVID-19 versus healthy individuals, *P* = 0.24; long COVID-19 versus acute COVID-19, *P* = 0.12; acute COVID-19 versus healthy individuals, *P* = 0.052; long COVID-19 versus non-PASC, *P* = 0.31; and non-PASC versus healthy individuals, *P* = 0.89).

### Sensitivity analysis

**Fig. 9:**
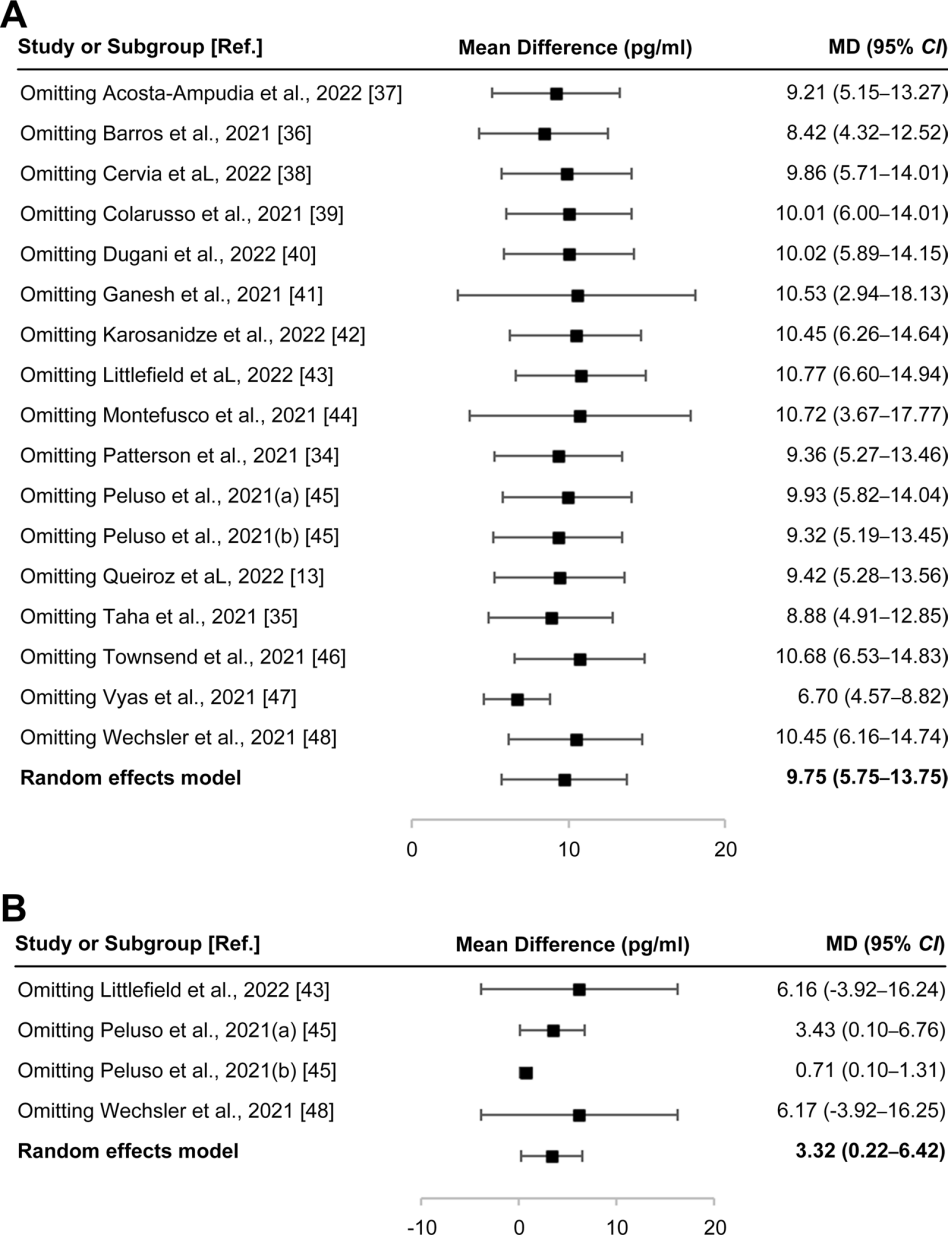
Sensitivity analysis of the association between pooled estimates of IL-6 levels and long COVID-19. **A** Sensitivity analysis for long COVID-19 vs healthy controls. **B** Sensitivity analysis for long COVID-19 vs non-PASC. Ref.: reference; MD: mean difference

The stability of the pooled results of the association between IL-6 levels and long COVID-19 was tested using a sensitivity analysis by excluding each individual study. As a result, no single study influenced the significance of either the pooled estimate of IL-6 (Fig. 3) or the comparison of pooled IL-6 estimates for long COVID-19 patients and healthy individuals or those without PASC (Fig. 9). These data suggest the reliability of the results obtained.

## Discussion

In normal individuals, IL-6 levels were estimated at 5.186 pg/ml [27]. IL-6 has been identified to be associated with several infections, including COVID-19 [16–18]. Increased levels of IL-6 were found to be significantly associated with adverse clinical COVID-19 outcomes such as ICU admission, acute respiratory distress syndrome (ARDS), and death. In addition, patients with such complicated forms of COVID-19 had nearly threefold higher serum IL-6 levels than those with noncomplicated disease [34–36].

An increase in the levels of IL-6 not only occurs in the acute infection period but is also one of the most critical factors that contributes to post-COVID-19 syndrome after a time window of four weeks post infection [41]. In this study, we demonstrated that increased IL-6 is associated with long COVID-19, as serum levels of IL-6 were found to be significantly elevated in patients after COVID-19 infection, whether in the acute or long COVID-19 phase. Such an informative finding aligns with previous studies summarized by Coomes and colleagues [49]. In addition, our data serve as a basic determinant for long COVID-19 in association with high levels of the immune mediator IL-6.

The sharp increase in IL-6 levels during acute infection is due to a cytokine storm [50], while it might decrease due to the different dynamic changes in IL-6 and antibodies during the long COVID-19 phase [51]. According to the New Coronavirus Pneumonia Prevention and Control Program (7th edition), a decreasing level of IL-6 indicates aggravation of COVID-19 [52]. Similarly, in this study, according to the forest plot results, IL-6 levels were found to be high in patients with long COVID-19 compared with healthy individuals and those without PASC. In addition, patients in the acute COVID-19 phase showed a higher level of IL-6 than those with long COVID-19. IL-6 has been shown to be a very sensitive indicator for monitoring infection and prognosis. In PASC, a significant increase in IL-6 levels [12] and a trend toward higher levels of IL-6 in early recovery [45] have been observed. Recent studies have shown that IL-6 and granulocyte–macrophage colony stimulating factor (GM-CSF) can be secreted by active pathogenic T cells upon SARS-CoV-2 infection. Additionally, CD14 + CD16 + inflammatory monocytes activated by GM-CSF could secrete more IL-6 and other inflammatory factors [53]. These results suggest that high levels of IL-6 could predict long COVID-19 or at least could inform the early status of long COVID-19.

This study revealed that increased IL-6 is associated with long COVID-19; however, the study could not assess from the current available data whether there are differences in such an association according to sex and age or whether it would be affected by a specific comorbidity. Littlefield and colleagues suggested that there was no difference in the levels of IL-6 in female and male PASC participants [43]. It has also been reported that elderly individuals have higher levels of IL-6 after acute infection [43] and that immunodeficiency in elderly individuals affects innate immunity [54]. Patients with comorbidities such as lung fibrotic-like changes have higher IL-6 levels [39]. The designs of these studies were correlational; therefore, the mechanisms underlying the relationship between IL-6 and several aspects of biodata and clinical features of the patients should be investigated in the future.

## Conclusions

We demonstrated that increased IL-6 is associated with long COVID-19. This study suggests a mean value of IL-6 estimated at 20.92 pg/ml for long COVID-19. Collectively, findings from this study suggest high levels of this immune mediator as a basic determinant for long COVID-19, which could serve as a predictor of long COVID-19 or at least could inform on the “early stage” of long COVID-19. However, it is unclear whether comorbidities facilitate an increase in the levels of IL-6 in COVID-19 subjects. Exploratory studies need to be conducted in this regard in the future.

## Supplementary Information

**Additional file 1.** Details of included studies.

**Additional file 2.** PRISMA checklist.

## Data Availability

The data analysed during this study are included in this published article and Additional files 1 and 2. All the datasets generated in this study are available following manuscript publication upon request from the corresponding author Kokouvi Kassegne (kassegnek@sjtu.edu.cn).

## Abbreviations

AIDS: Acquired immune deficiency syndrome
ARDS: Acute respiratory distress syndrome
CDC: Centre for Disease Control and Prevention
*CI*: Confidence interval
COVID-19: Coronavirus disease 2019
EV71: Enterovirus 71
ICU: Intensive care unit
IL-1β: Interleukin-1 beta
IQR: Interquartile range
IL-6: Interleukin-6
NICE: National Institute for Health and Care Excellence, United States of America
NIH: National Institute of Health, United States of America
NOS: New Ottawa scale
PASC: Postacute sequelae of COVID-19
PRISMA: Preferred Reporting Items for Systematic Reviews and Meta-Analyses
SARS-CoV-2: Severe acute respiratory syndrome coronavirus 2
*SD*: Standard deviation
TNF-α: Tumour necrosis factor α
WHO: World Health Organization
